# An investigation of the pattern and mechanism of comorbidity in patients with Hashimoto’s thyroiditis

**DOI:** 10.3389/fendo.2025.1615095

**Published:** 2025-08-25

**Authors:** Caihong Zhao, Haodong Xiong, Lingfei Zhu, Azijiang Adali, Weijie Yu, Simiao Tan, Shuying Wang, Chengbowen Zhao, Yan Lin, Zinan Wei, He Huang, Xinyu Peng

**Affiliations:** ^1^ Dongzhimen Hospital, Beijing University of Chinese Medicine, Beijing, China; ^2^ School of Life Sciences, Beijing University of Chinese Medicine, Beijing, China; ^3^ School of Traditional Chinese Medicine, Beijing University of Chinese Medicine, Beijing, China

**Keywords:** Hashimoto’s thyroiditis, comorbidities, association rules, pathogenesis, network pharmacology

## Abstract

**Objective:**

This study aimed to investigate comorbidity patterns and potential pathogenic mechanisms in patients with Hashimoto’s thyroiditis (HT).

**Methods:**

Patients with HT who visited the outpatient clinic of the Thyroid Department at Dongzhimen Hospital, Beijing University of Chinese Medicine, between June 2021 and December 2024 were included. Association rule analysis and logistic regression analysis were performed using SPSS 25.0 and SPSS Modeler 18.0 to identify comorbidity patterns. Disease targets were screened using the GeneCards database, and protein interaction networks for intersecting targets were constructed using STRING and Cytoscape. GO function and KEGG pathway enrichment analyses were performed with Metascape to uncover relevant targets and potential pathways associated with comorbidities in patients with HT.

**Results:**

Among 429 patients with HT, 348 had comorbidities, resulting in a comorbidity prevalence of 81.19%. Association rule analysis identified thyroid nodules (TN) as the core binary comorbidity. The combination of TN and hyperplasia of the mammary gland (HMG) was central to ternary comorbidities, while the trio of TN, HMG, and uterine leiomyomas (UL) characterized quaternary comorbidities. Being a woman and advancing age were associated with increased risk of comorbidities, whereas levothyroxine sodium (L-T4) therapy was linked to reduced risk. Core targets associated with comorbidity prediction included AKT1, TP53, EGFR, INS, and TNF. Key pathways involved were the cancer pathway and PI3K–Akt signaling pathway.

**Conclusion:**

The high prevalence of comorbidities in patients with HT warrants increased clinical attention within the medical community.

## 1 Introduction

Thyroid nodules (TN), hyperplasia of the mammary gland (HMG), uterine leiomyomas (UL), and benign breast nodules (BN) frequently occur either simultaneously or sequentially, leading to multisystem disease presentations that have garnered significant scholarly attention. It has been reported that patients with UL have a 38.7% likelihood of developing TN, compared to just 20% in the general population, suggesting that UL may elevate the risk of TN (1). Additionally, patients with BN are more prone to concomitant TN, and vice versa, indicating a bidirectional pathogenic relationship between BN and TN (2). Some researchers refer to TN, HMG, and UL as the “female triad,” implying that the presence of nodules, hyperplasia, or masses in any one of these organs (thyroid, breast, or uterus) in female patients often correlates with accompanying symptoms in the other two. This points to shared causative factors and pathogenic mechanisms among these diseases (3). Recent studies have revealed that patients with Hashimoto’s thyroiditis (HT) frequently present with multiple comorbidities (4, 5), with TN co-occurrence rates as high as 24.3% (6) and cancer complication rates reaching 58.3% (7–9). In clinical practice, it is common for patients with HT to be diagnosed with various systemic diseases; however, the specific comorbidities, influencing factors, and underlying pathogenesis remain unclear. Therefore, this study aims to explore the comorbidity patterns, related influencing factors, and potential pathogenesis in patients with HT, thereby offering valuable insights for clinical diagnosis and treatment.

## 2 Information and methodology

### 2.1 Subject of the study

A total of 429 patients with HT admitted to the Thyroid Department at Dongzhimen Hospital, Beijing University of Chinese Medicine, between June 2021 and December 2024, were included in the study. The study adhered to ethical principles, and ethical approval was obtained (Approval No. 2018BZHYLL0303).

### 2.2 Diagnostic criteria

The diagnostic criteria for HT were based on the Chinese Guidelines for the Diagnosis and Treatment of Thyroid Diseases—Thyroiditis (10).

### 2.3 Inclusion and exclusion criteria

#### 2.3.1 Inclusion criteria

(1) Fulfillment of the diagnostic criteria for HT; (2) Age between 18 and 70 years; (3) Presence of thyroid function abnormalities; (4) Informed consent obtained.

#### 2.3.2 Exclusion criteria

(1) Patients with mental disorders; (2) Pregnant or breastfeeding individuals; (3) Individuals with severe cardiac, hepatic, or renal diseases; (4) Individuals with malignant tumors.

### 2.4 Sample size calculation

This study was cross-sectional, and the sample size was calculated using the formula N = (Z_α/2_)^2^P(1-P)/d^2^, where Z_α/2_ is the Z value of the normal distribution, P is the prevalence rate, and d is the permissible error. For this study, α = 0.05, Z_α/2_ = 1.96, and d = 0.1. Based on a prior study indicating a 24.3% prevalence of TN in patients with HT, P was set to 0.243. Accounting for a 10% dropout rate, at least 79 patients with HT were required, and 429 patients were included, which fulfilled the sample size requirement.

### 2.5 Observation indicators

#### 2.5.1 General information

General information included sex (man, woman); age (continuous variable); marital status (married, unmarried); education level (junior high school or below, junior or senior high school, undergraduate, postgraduate and above); and body mass index (BMI: < 18.5 kg/m^2^, 18.5 ≤ BMI < 24.0 kg/m^2^, 24.0 ≤ BMI < 28.0 kg/m^2^, BMI ≥ 28.0 kg/m^2^).

#### 2.5.2 Daily life behaviors

Daily life behaviors included smoking (defined as having smoked at least 100 cigarettes in one’s lifetime) and alcohol consumption (defined as consuming at least 12 alcoholic beverages per year).

#### 2.5.3 Psychosomatic situation

The psychosomatic situation included a history of stress (defined as experiencing the death of a loved one or a major family change within the past year). Anxiety and depression were also evaluated. Anxiety was defined as a symptom of emotional disturbance characterized by a fear of personal safety and undesirable outcomes, assessed based on the respondent’s self-perception. Depression was described as a common mental disorder characterized by a lack of clear goals and mental disillusionment, and was also assessed through self-perception.

#### 2.5.4 Disease history

Medical conditions considered included TN, HMG, BN, UL, gallbladder polyps (GBP), pulmonary nodules (PN), gastric polyps (GP), and intestinal polyps (IP) were considered. These conditions were identified based on self-reported physician diagnoses or relevant ultrasound findings.

#### 2.5.5 Thyroid autoantibody testing

Thyroid peroxidase antibody (TPOAb) positivity was defined as TPOAb > 9.0 IU/ml, and thyroglobulin antibody (TGAb) positivity was defined as TGAb > 115.0 IU/ml.

#### 2.5.6 History of treatment for thyroid disease

The history of treatment included the use of levothyroxine sodium (L-T4) and methimazole tablets.

### 2.6 Core target acquisition and pathway enrichment

#### 2.6.1 Core targets of comorbidities in patients with HT

Disease targets for HT, TN, HMG, UL, and BN were retrieved from the GeneCards database (https://www.genecards.org/). The VLOOKUP function in Excel 2019 was used to identify intersecting disease targets, which were then imported into the STRING database (https://cn.string-db.org/) to construct a protein–protein interaction (PPI) network. Cytoscape 3.7 was used to visualize the top 50 targets based on DEGREE rankings and identify core targets.

#### 2.6.2 Pathway enrichment for comorbidities in patients with HT

Gene Ontology (GO) function and Kyoto Encyclopedia of Genes and Genomes (KEGG) pathway enrichment analyses of the intersecting targets were conducted using the Metascape database (https://metascape.org/), with the results visualized via the microbiome platform.

### 2.7 Statistical analyses

For data analysis, Excel 2019, SPSS Modeler 18.0, and Cytoscape 3.7 were used to perform frequency analysis, association rule analysis, and complex network visualization to investigate comorbidity patterns in patients with HT. SPSS 25.0 was used for both univariate and multivariate logistic regression analyses to identify factors influencing comorbidity. Categorical data were presented as frequency (n) or proportion (%), and Pearson’s chi-square test or Fisher’s exact test was applied for group comparisons. For continuous data not following a normal distribution, the median (P25, P75) was reported, and the Mann–Whitney U test was used for between-group comparisons. Statistical significance was set at *p* < 0.05.

## 3 Results

### 3.1 Analysis of comorbidities in patients with HT

A total of eight types of comorbidities were identified among the 429 patients with HT. The most prevalent comorbidities were TN, HMG, UL, and BN, accounting for 53.1%, 24.9%, 24.2%, and 23.5% of cases, respectively, as detailed in **Table 1**.

**Table 1:** Comorbidities in patients with HT.

Disease names for comorbidities	Numbers(%)
TN/case (%)	228 (53.1%)
HMG/case (%)	203 (47.3%)
UL/case (%)	104 (24.2%)
BN/case (%)	101 (23.5%)
GBP/case (%)	23 (5.3%)
PN/case (%)	19 (4.4%)
GP/case (%)	13 (3.0%)
IP/case (%)	3 (0.6%)

### 3.2 Analysis of comorbidity patterns in patients with HT

Among the 429 patients with HT, 348 presented with comorbidities, resulting in a prevalence of 81.19%. Of these, 137 patients had one comorbidity, 110 had two, 73 had three, and 28 had four or more comorbidities, as shown in **Table 2**.

**Table 2:** Patterns of comorbidity in patients with HT.

Patterns of comorbidity	Numbers (%)	Patterns of comorbidity	Numbers (%)
Single HT/case (%)	81 (18.9%)	TN + HMG + GBP	5
1 type of disease/case (%)	137 (31.9%)	TN + HMG + PN	2
TN	58	TN + HMG + GP	2
HMG	51	TN + UL + BN	5
UL	10	TN + UL + GBP	1
BN	14	TN + UL + GP	2
GBP	2	TN + BN + GBP	2
PN	1	TN + BN + PN	1
IP	1	TN + GBP + PN	1
2 types of disease/case (%)	110 (25.6%)	HMG + UL + BN	2
TN + HMG	39	HMG + UL + GBP	1
TN + UL	11	HMG + UL + PN	2
TN + BN	20	HMG + BN + GBP	1
TN + GBP	2	4 and more types of diseases/case (%)	28 (6.5%)
TN + PN	2	TN + HMG + UL + BN	9
TN + GP	1	TN + HMG + UL + PN	4
TN + IP	1	TN + BN + UL + GP	4
HMG + UL	13	TN + BN + UL + GBP	1
HMG + BN	11	TN + UL + BN + GBP	1
HMG + GBP	3	TN + UL + BN + PN	2
HMG + GP	1	TN + UL + BN + IP	1
UL + BN	4	HMG + UL + BN + PN	1
BN + PN	1	TN + HMG + UL + BN + GBP	1
GBP + GP	1	TN + HMG + UL + BN + PN	1
3 types of disease/case (%)	73 (17.0%)	TN + HMG + UL + BN + GP	2
TN + HMG + UL	26	TN + HMG + UL + BN + GBP + PN	1
TN + HMG + BN	20		

HT cases grouped by comorbidity count (0=Single; 1-3=number-specific; ≥4=multimorbid) from 8 predefined conditions (TN, HMG, UL, BN, GBP, PN, IP).

Single HT:HT patients without comorbidities: 1 type of disease: HT patients with 1 comorbidity: 2 types of disease: HT patients with 2 comorbidities: 3 types of disease: HT patients with 3 comorbidities: 4 and more types of disease: HT patients with ≥4 comorbidities.

### 3.3 Association rule analysis of co-morbid conditions in patients with HT

By setting the minimum conditional support to 10, the minimum confidence level to 10, and the maximum number of antecedents to five, 29 strong association rules were identified. These included 13 strong rules for binary comorbidity patterns, with TN being the most frequent; 13 strong rules for ternary comorbidity patterns, with TN and HMG as the most frequent combination; and three strong rules for quaternary comorbidity patterns, with TN, HMG, and UL being the most common combination. Detailed results are presented in **Table 3**.

**Table 3:** Association rule analysis of co-morbid conditions in patients with HT.

Posterior term	Preterm	Support	Confidence	Enhancement	Numbers
TN	BN	23.54	66.34	1.25	101
UL	BN	23.54	29.70	1.23	101
HMG	BN	23.54	49.50	1.05	101
TN	UL	24.24	68.27	1.28	104
HMG	UL	24.24	64.42	1.36	104
BN	UL	24.24	28.85	1.23	104
PN	UL	24.24	10.58	2.39	104
TN	HMG	47.32	57.64	1.08	203
BN	HMG	47.32	24.63	1.05	203
UL	HMG	47.32	33.00	1.36	203
HMG	TN	53.15	51.32	1.08	228
BN	TN	53.15	29.39	1.25	228
UL	TN	53.15	31.14	1.28	228
TN	BN + HMG	11.66	70.00	1.32	50
UL	BN + HMG	11.66	34.00	1.40	50
TN	UL + HMG	15.62	71.64	1.35	67
HMG	BN + TN	15.62	52.24	1.10	67
UL	BN + TN	15.62	34.33	1.42	67
BN	UL + HMG	15.62	25.37	1.08	67
PN	UL + HMG	15.62	13.43	3.03	67
HMG	UL+ TN	16.55	67.61	1.43	71
BN	UL + TN	16.55	32.39	1.38	71
GP	UL + TN	16.55	11.27	3.72	71
PN	UL + TN	16.55	11.27	2.54	71
UL	HMG + TN	27.27	41.03	1.69	117
BN	HMG + TN	27.27	29.91	1.27	117
BN	UL + HMG + TN	11.19	29.17	1.24	48
GP	UL + HMG + TN	11.19	12.50	4.13	48
PN	UL + HMG + TN	11.19	12.50	2.82	48

### 3.4 Analysis of factors influencing comorbidities in patients with HT

#### 3.4.1 One-way logistic regression analysis

One-way logistic regression analysis revealed statistically significant differences between the comorbidity and non-comorbidity groups in terms of sex, age, marital status, smoking history, TPOAb status, and TGAb status (*p* < 0.05), as presented in **Table 4**.

**Table 4:** One-way logistic regression analysis.

Items	Single HT group (n = 81)	Comorbidity group (n = 348)	*x* ^2^/*F/z*	*p*
Sex			23.881	<0.001
Male patients	14 (17.3%)	11 (3.2%)		
Female patients	67 (82.7%)	337 (96.8%)		
Age (years)	34.00 (27.00,45.50)	42.00 (33.00,52.75)	-4.622	<0.001
Marriage			15.183	<0.001
Unmarried	32 (39.5%)	67 (19.3%)		
Married	49 (60.5%)	281 (80.7%)		
Educational level			2.362	0.501
Junior high school and below	2 (2.5%)	21 (6.0%)		
Secondary/High School	10 (12.3%)	48 (13.8%)		
College/Undergraduate	57 (70.4%)	220 (63.2%)		
Postgraduate and above	12 (14.8%)	59 (17.0%)		
BMI (kg/m2)			3.661	0.300
BMI < 18.5	9 (11.1%)	21 (6.0%)		
18.5 ≤ BMI <24.0	46 (56.8%)	220 (63.2%)		
24.0 ≤ BMI <28.0	17 (21.0%)	79 (22.7%)		
BMI ≥ 28.0	9 (11.1%)	28 (8.0%)		
Smoking history			4.012	0.045
NO	73 (90.1%)	333 (95.7%)		
YES	8 (9.9%)	15 (4.3%)		
Drinking history			3.193	0.074
NO	69 (85.2%)	319 (91.7%)		
YES	12 (14.8%)	29 (8.3%)		
Stress history			0.246	0.620
NO	64 (79.0)	266 (76.4%)		
YES	17 (21.0%)	82 (23.6%)		
Anxiety states (times)			0.350	0.554
< 3 per week	24 (29.6%)	115 (33.0%)		
≥ 3 per week	57 (70.4%)	233 (67.0%)		
Depressive state (times)			0.488	0.485
< 3 per week	47 (58.0%)	187 (53.7%)		
≥ 3 per week	34 (42.0%)	161 (46.3%)		
TPOAB, TGAB status			4.172	0.044
single antibody positive	24 (29.6%)	146 (42.0%)		
both antibodies positive	57 (70.4%)	202 (58.0%)		
History of taking L-T4			4.758	0.029
NO	54 (66.7%)	272 (78.2%)		
YES	27 (33.3%)	76 (21.8%)		
History of taking methimazole tablets.			0.002	0.960
NO	76 (93.8%)	326 (93.7%)		
YES	5 (6.2%)	22 (6.3%)		

#### 3.4.2 Multifactorial logistic regression analysis

Multifactorial logistic regression analysis identified statistically significant differences between the two groups in terms of sex, age, and L-T4 treatment history (*p* < 0.05), as shown in **Tables 5**, **6**.

**Table 5:** Variable assignment table.

Variable names	Assignment
Sex	Male patient = 1, Female patient = 2
Age	continuous variable
Marriage	Unmarried=1, Married = 2
TPOAB, TGAB status	single antibody positive = 1, both antibodies positive = 2
History of taking L-T4	NO = 1, YES = 2

**Table 6:** Results of binary logistic regression analysis of comorbidities in patients with HT.

Variable names	β	SE	x^2^	*p*	OR	95%CI
Sex (reference: male)	1.831	0.443	17.120	<0.001	6.242	2.622-14.861
Age (continuous variable)	0.038	0.014	6.956	0.008	1.039	1.010-1.069
Marriage (reference: unmarried)	0.561	0.355	2.501	0.114	1.753	0.874-3.514
TPOAB, TGAB status (reference: single antibody positive)	-0.448	0.281	2.462	0.117	0.639	0.365-1.118
History of taking L-T4 (reference: No)	-0.867	0.296	8.580	0.003	0.420	0.235-0.751
constant	-1.599	0.663	5.807	0.016	0.202	

### 3.5 Pathogenesis of comorbidities in patients with HT

#### 3.5.1 Potential core targets of HT comorbidities

**Figure 1:**
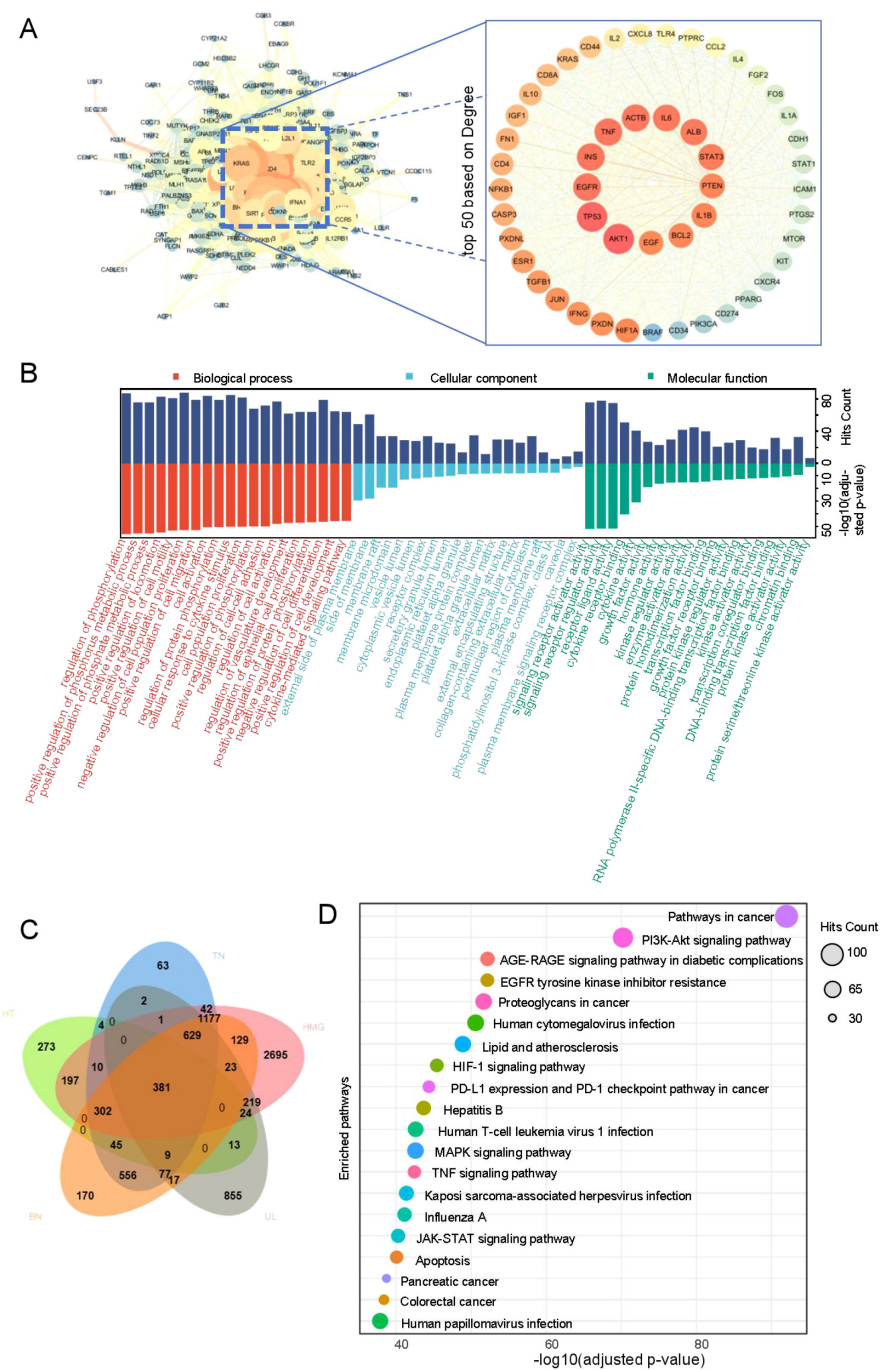
Core target and pathway enrichment.

The most common co-morbid conditions in patients with HT were TN, HMG, UL, and BN. These four diseases were selected for the investigation of potential pathogenesis in HT-related comorbidity. A total of 1,258 HT targets, 3,298 TN targets, 5,829 HMG targets, 2,250 UL targets, 3,515 BN targets, and 381 intersecting targets across the five diseases were obtained from the GeneCards database. The intersecting targets were imported into the STRING database to construct a PPI network. The network was analyzed using Cytoscape 3.7.2 to visualize the top 50 targets based on DEGREE ranking (**Figures 1A, C**). The results suggest that core targets associated with comorbidities in patients with HT may include AKT1, TP53, EGFR, INS, and TNF.

#### 3.5.2 Potential pathway enrichment for comorbidities in patients with HT

Using the Metascape database, with parameters set to Homo sapiens, count ≥ou and *p* < 0.01, a total of 2,813 biological processes (BP), 294 molecular functions (MF), 121 cellular components (CC), and 208 pathways were identified. The top two enriched pathways were the cancer pathway and the PI3K–Akt signaling pathway. The top 20 enriched BP, CC, MF, and KEGG pathways, selected based on adjusted *p*-values, were visualized as bubble diagrams using the microbiome platform (**Figures 1B, D**), with detailed results provided in **Supplementary Table 1**.

## 4 Discussion

Among the 429 patients with HT, 348 presented with comorbidities, resulting in a prevalence rate of 81.19%. Eight types of comorbidities were identified, with TN, HMG, UL, and BN being the most prevalent, accounting for 53.1%, 24.9%, 24.2%, and 23.5% of cases, respectively. Association rule analysis revealed that TN was central to the binary comorbidity pattern, while TN and HMG formed the core of the ternary pattern, and TN, HMG, and UL were central to the quaternary pattern. These findings highlight the high prevalence of comorbidities in patients with HT and emphasize the need to explore contributing factors and potential pathogenesis.

Regarding influencing factors, univariate analysis identified sex, age, marital status, TPOAb/TGAb antibody status, and L-T4 treatment history as significant correlates of HT comorbidity. Multivariate logistic regression further confirmed that sex, age, and L-T4 treatment history were independently associated with HT comorbidity. Specifically, the likelihood of comorbidities was significantly higher in women and older patients with HT. Female patients with HT were found to be 6.242 times more likely to have comorbidities than their male counterparts, potentially due to greater variability in estrogen levels (11). Estrogen receptors are present in the tissues of the thyroid, breast, and uterus, suggesting that fluctuations in estrogen may play a significant role in the development of comorbidities in female patients with HT (12–14). Previous studies have also linked increasing age with the incidence of nodular disorders (15), a finding consistent with our results.

Interestingly, patients with HT undergoing L-T4 therapy had a lower likelihood of developing TN, breast hyperplasia, mammary nodules, and uterine fibroids. This could be attributed to L-T4 treatment correcting the hypothyroid state and the associated endocrine and metabolic disturbances. Patients with HT often experience hypothyroidism, which leads to elevated TSH levels that can stimulate thyroid tissue hyperplasia and nodule formation, increasing the risk of benign TN (16). L-T4 therapy helps normalize thyroid hormone levels and reduce TSH, thereby decreasing overstimulation of thyroid follicular cells, lowering the risk of hyperplasia and nodules, and potentially causing existing nodules to regress (17–20).

Additionally, thyroid hormone levels interact with estrogen (21, 22), and chronic exposure to elevated estrogen has been linked to a higher susceptibility to hypothyroidism (23). High estrogen levels can also influence the normal development of the mammary glands and uterus (24). Furthermore, metabolic disorders such as insulin resistance, lipid metabolism disturbances, and chronic inflammation—often associated with hypothyroidism—may indirectly affect cell proliferation and increase tumor risk (25).

PPI network analysis identified AKT1, TP53, EGFR, INS, and TNF as potential core targets for comorbidities in patients with HT. To determine whether these targets were abnormally expressed in patients with HT, GEO data (GSE138198), which profile mRNA expression in the thyroid tissues of patients with HT, were analyzed alongside relevant literature. The GEO data revealed the expression of AKT1, TP53, EGFR, and TNF mRNAs in the thyroid tissues of patients with HT, with TP53 showing significantly higher expression (*p*_adj_ < 0.05; **Supplementary Figure 1**). Literature reports also confirmed that TP53, which encodes the cellular tumor antigen P53 (P53), is overexpressed in the thyroid gland of patients with HT (26).

In contrast, the GEO data did not show significant differences in the mRNA expression of AKT1, EGFR, TNF, and INS in the thyroid tissues of patients with HT. This may be attributed to factors such as mRNA translation efficiency, post-translational modifications, and external influences on protein expression. Notably, in human normal thyroid cells (Nthy-ori 3-1), phosphorylation of AKT (p-AKT) is significantly upregulated in response to IL-23 stimulation, a cytokine known to be elevated in patients with HT (27). EGFR expression has also been reported to be elevated in the thyroid tissues of patients with HT (28). INS, secreted by pancreatic islet cells, often accompanies an increase in insulin secretion in patients with HT (29). Furthermore, TNF-α is significantly elevated in the serum of patients with HT (30). Thus, AKT1, EGFR, TNF, and INS demonstrate characteristically high expression in the thyroid tissues, cells, or peripheral blood of patients with HT.

Mechanistically, AKT1 is a critical component of the PI3K/Akt signaling pathway, which plays a vital role in cell proliferation, survival, and metabolism (31). Activated AKT promotes the survival and proliferation of thyroid follicular epithelial cells, breast ductal epithelial cells, and uterine smooth muscle cells, inhibits apoptosis, and supports the formation of nodules and leiomyomas (32–34). High expression of AKT or p-AKT has been linked to the development of TN, HMG, and UL (35–37). TP53, a well-known tumor suppressor gene, is frequently mutated in various cancers, including breast and thyroid cancers (38). Literature indicates that TP53 is also overexpressed in benign TN, HMG, and UL (26, 39, 40). EGF stimulates cell proliferation by binding to its receptor EGFR, which activates downstream MAPK and PI3K-Akt signaling pathways (41). Recent studies have demonstrated that EGFR signaling intersects extensively with inflammatory factor signaling, creating a positive feedback loop that amplifies both pro-proliferative and pro-inflammatory signals (42). Elevated EGFR expression levels have been observed in the diseased tissues of patients with both benign and malignant TN (43), where it promotes abnormal breast tissue proliferation (44) and UL development (45)INS, a major regulator of glucose metabolism, also has potent mitogenic and anti-apoptotic effects. By activating the PI3K/Akt pathway through the insulin receptor, it regulates cell metabolism and growth (46). Both TN and UL have been associated with insulin resistance, although the relationship between HMG and INS remains unclear (47–49). TNF, a pro-inflammatory cytokine, activates the PI3K/Akt pathway and exacerbates immune-inflammatory responses (50). High TNF expression has been reported in TN, HMG, and UL.

This study focused on the comorbidity patterns and potential mechanisms in patients with HT, integrating clinical data, statistical modeling, network pharmacology, and pathway enrichment analysis. The findings suggest that the TN–HMG–UL triad constitutes the core comorbidity cluster in HT. Being a woman and increasing age are associated with a higher likelihood of comorbidities, while L-T4 administration is linked to a decreased likelihood. AKT1, TP53, EGFR, INS, TNF, and the PI3K–Akt signaling pathway were identified as potential molecular foundations for HT-related co-morbidities.

Notably, this study employed a cross-sectional design, which allows for the identification of clinical epidemiological characteristics related to HT comorbidities but does not establish causal relationships. Further verification through multicenter, large-sample cohort studies is necessary. Additionally, network pharmacology was used to predict potential core targets of HT-related comorbidities. Preliminary support for the actual expression of these targets in the tissues of patients with HT was provided by the GEO database and relevant literature. However, due to the study’s limitations, only literature cross-references were used to support the functional relevance of these targets. Future clinical validations—such as HT co-morbidomics—are required to provide direct evidence for the prevention, diagnosis, and treatment of the disease.

## Data Availability

The raw data supporting the conclusions of this article will be made available by the authors, without undue reservation.
