# Supplementary material for: An investigation of the pattern and mechanism of comorbidity in patients with Hashimoto’s thyroiditis
Source: Front Endocrinol (Lausanne). 2025 Aug 25;16:1615095. doi: 10.3389/fendo.2025.1615095 (PMC12415523; doi:10.3389/fendo.2025.1615095)
Supplement: Supplementary file 1 [file DataSheet1.docx]

Supplementary Table 1 GO and KEGG pathway enrichment analysis

| **Category** | **Terms** | **LogP** | **Log(adjusted p-value)** | **Enrichment** | **Z-score** |
| --- | --- | --- | --- | --- | --- |
| Biological process | GO:0042325:regulation of phosphorylation | -60.05 | -55.87 | 9.96 | 26.97 |
| Biological process | GO:0010562:positive regulation of phosphorus metabolic process | -58.96 | -55.27 | 12.25 | 28.44 |
| Biological process | GO:0045937:positive regulation of phosphate metabolic process | -58.96 | -55.27 | 12.25 | 28.44 |
| Biological process | GO:0040017:positive regulation of locomotion | -58.02 | -54.45 | 10.21 | 26.72 |
| Biological process | GO:2000147:positive regulation of cell motility | -56.58 | -53.11 | 10.22 | 26.40 |
| Biological process | GO:0008285:negative regulation of cell population proliferation | -56.11 | -52.72 | 8.80 | 25.15 |
| Biological process | GO:0030335:positive regulation of cell migration | -55.94 | -52.61 | 10.47 | 26.43 |
| Biological process | GO:0001775:cell activation | -53.65 | -50.39 | 8.86 | 24.66 |
| Biological process | GO:0001932:regulation of protein phosphorylation | -53.53 | -50.32 | 9.75 | 25.34 |
| Biological process | GO:0071345:cellular response to cytokine stimulus | -53.22 | -50.05 | 8.58 | 24.34 |
| Biological process | GO:0008283:cell population proliferation | -53.17 | -50.04 | 9.08 | 24.73 |
| Biological process | GO:0042327:positive regulation of phosphorylation | -52.84 | -49.75 | 12.37 | 27.02 |
| Biological process | GO:0022407:regulation of cell-cell adhesion | -52.78 | -49.73 | 11.16 | 26.19 |
| Biological process | GO:0050865:regulation of cell activation | -50.82 | -47.80 | 9.37 | 24.41 |
| Biological process | GO:1901342:regulation of vasculature development | -49.95 | -46.95 | 13.25 | 26.83 |
| Biological process | GO:0050678:regulation of epithelial cell proliferation | -49.80 | -46.84 | 12.44 | 26.29 |
| Biological process | GO:0001934:positive regulation of protein phosphorylation | -49.46 | -46.52 | 12.29 | 26.10 |
| Biological process | GO:0045596:negative regulation of cell differentiation | -48.94 | -46.02 | 8.50 | 23.29 |
| Biological process | GO:0010720:positive regulation of cell development | -48.51 | -45.61 | 11.56 | 25.38 |
| Biological process | GO:0019221:cytokine-mediated signaling pathway | -48.33 | -45.46 | 11.80 | 25.50 |
| Cellular component | GO:0009897:external side of plasma membrane | -32.72 | -29.44 | 9.71 | 19.83 |
| Cellular component | GO:0098552:side of membrane | -30.94 | -27.96 | 6.53 | 17.23 |
| Cellular component | GO:0045121:membrane raft | -22.08 | -19.30 | 9.29 | 16.03 |
| Cellular component | GO:0098857:membrane microdomain | -21.99 | -19.30 | 9.22 | 15.97 |
| Cellular component | GO:0044853:plasma membrane raft | -9.65 | -7.60 | 9.68 | 10.52 |
| Cellular component | GO:0005901:caveola | -6.07 | -4.37 | 8.83 | 7.97 |
| Cellular component | GO:0031983:vesicle lumen | -15.52 | -12.93 | 6.99 | 12.34 |
| Cellular component | GO:0060205:cytoplasmic vesicle lumen | -14.65 | -12.14 | 6.76 | 11.87 |
| Cellular component | GO:0034774:secretory granule lumen | -13.03 | -10.65 | 6.38 | 10.99 |
| Cellular component | GO:0031091:platelet alpha granule | -11.06 | -8.78 | 12.23 | 12.11 |
| Cellular component | GO:0031093:platelet alpha granule lumen | -10.37 | -8.17 | 14.24 | 12.24 |
| Cellular component | GO:0043235:receptor complex | -13.72 | -11.28 | 4.99 | 10.57 |
| Cellular component | GO:0098797:plasma membrane protein complex | -10.66 | -8.42 | 3.78 | 8.62 |
| Cellular component | GO:0098802:plasma membrane signaling receptor complex | -4.72 | -3.15 | 3.66 | 5.45 |
| Cellular component | GO:0005788:endoplasmic reticulum lumen | -12.45 | -10.12 | 6.31 | 10.69 |
| Cellular component | GO:0031012:extracellular matrix | -10.29 | -8.14 | 4.20 | 8.69 |
| Cellular component | GO:0030312:external encapsulating structure | -10.28 | -8.14 | 4.19 | 8.67 |
| Cellular component | GO:0062023:collagen-containing extracellular matrix | -10.21 | -8.10 | 4.78 | 8.94 |
| Cellular component | GO:0048471:perinuclear region of cytoplasm | -9.90 | -7.82 | 3.63 | 8.20 |
| Cellular component | GO:0005943:phosphatidylinositol 3-kinase complex, class IA | -9.51 | -7.50 | 52.99 | 17.61 |
| Molecular function | GO:0030546:signaling receptor activator activity | -55.43 | -51.73 | 11.02 | 26.73 |
| Molecular function | GO:0030545:signaling receptor regulator activity | -54.88 | -51.48 | 10.37 | 26.12 |
| Molecular function | GO:0048018:receptor ligand activity | -54.66 | -51.44 | 11.02 | 26.54 |
| Molecular function | GO:0005126:cytokine receptor binding | -43.40 | -40.30 | 14.74 | 25.84 |
| Molecular function | GO:0005125:cytokine activity | -33.80 | -30.80 | 13.93 | 22.41 |
| Molecular function | GO:0008083:growth factor activity | -21.76 | -18.83 | 13.25 | 17.64 |
| Molecular function | GO:0070851:growth factor receptor binding | -16.13 | -13.50 | 12.01 | 14.68 |
| Molecular function | GO:0005179:hormone activity | -19.06 | -16.20 | 13.85 | 16.70 |
| Molecular function | GO:0019207:kinase regulator activity | -18.14 | -15.34 | 8.31 | 14.04 |
| Molecular function | GO:0008047:enzyme activator activity | -17.96 | -15.21 | 5.34 | 12.38 |
| Molecular function | GO:0019887:protein kinase regulator activity | -15.52 | -12.99 | 8.10 | 12.86 |
| Molecular function | GO:0019209:kinase activator activity | -14.42 | -11.98 | 10.74 | 13.41 |
| Molecular function | GO:0030295:protein kinase activator activity | -12.71 | -10.47 | 10.29 | 12.40 |
| Molecular function | GO:0043539:protein serine/threonine kinase activator activity | -4.42 | -2.97 | 7.62 | 6.39 |
| Molecular function | GO:0042803:protein homodimerization activity | -17.81 | -15.10 | 4.91 | 12.05 |
| Molecular function | GO:0008134:transcription factor binding | -17.20 | -14.53 | 5.37 | 12.12 |
| Molecular function | GO:0061629:RNA polymerase II-specific DNA-binding transcription factor binding | -14.88 | -12.40 | 6.60 | 11.89 |
| Molecular function | GO:0001221:transcription coregulator binding | -14.18 | -11.76 | 12.44 | 13.88 |
| Molecular function | GO:0140297:DNA-binding transcription factor binding | -13.46 | -11.16 | 5.21 | 10.59 |
| Molecular function | GO:0003682:chromatin binding | -11.46 | -9.25 | 4.27 | 9.23 |
| KEGG Pathways | hsa05200:Pathways in cancer | -94.54 | -91.98 | 15.66 | 38.54 |
| KEGG Pathways | hsa04151:PI3K-Akt signaling pathway | -72.46 | -70.20 | 17.13 | 34.84 |
| KEGG Pathways | hsa04933:AGE-RAGE signaling pathway in diabetic complications | -54.22 | -52.14 | 33.84 | 37.32 |
| KEGG Pathways | hsa01521:EGFR tyrosine kinase inhibitor resistance | -54.08 | -52.13 | 39.74 | 39.17 |
| KEGG Pathways | hsa05205:Proteoglycans in cancer | -53.49 | -51.63 | 20.65 | 31.79 |
| KEGG Pathways | hsa05163:Human cytomegalovirus infection | -52.35 | -50.57 | 18.99 | 30.65 |
| KEGG Pathways | hsa05417:Lipid and atherosclerosis | -50.57 | -48.86 | 19.14 | 30.19 |
| KEGG Pathways | hsa04066:HIF-1 signaling pathway | -47.05 | -45.39 | 28.90 | 33.09 |
| KEGG Pathways | hsa05235:PD-L1 expression and PD-1 checkpoint pathway in cancer | -45.94 | -44.34 | 32.68 | 33.97 |
| KEGG Pathways | hsa05161:Hepatitis B | -45.16 | -43.64 | 21.46 | 29.56 |
| KEGG Pathways | hsa05166:Human T-cell leukemia virus 1 infection | -44.01 | -42.57 | 17.03 | 27.19 |
| KEGG Pathways | hsa04010:MAPK signaling pathway | -43.96 | -42.55 | 14.04 | 25.63 |
| KEGG Pathways | hsa04668:TNF signaling pathway | -43.78 | -42.40 | 26.05 | 30.91 |
| KEGG Pathways | hsa05167:Kaposi sarcoma-associated herpesvirus infection | -42.70 | -41.35 | 18.25 | 27.35 |
| KEGG Pathways | hsa05164:Influenza A | -42.41 | -41.09 | 19.76 | 27.93 |
| KEGG Pathways | hsa04630:JAK-STAT signaling pathway | -41.54 | -40.24 | 19.87 | 27.69 |
| KEGG Pathways | hsa04210:Apoptosis | -41.31 | -40.04 | 22.96 | 28.87 |
| KEGG Pathways | hsa05212:Pancreatic cancer | -39.94 | -38.68 | 33.03 | 31.77 |
| KEGG Pathways | hsa05210:Colorectal cancer | -39.56 | -38.35 | 30.15 | 30.73 |
| KEGG Pathways | hsa05165:Human papillomavirus infection | -39.04 | -37.85 | 12.17 | 23.14 |


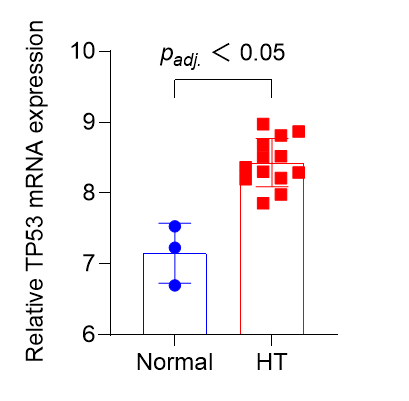


Supplementary figure 1. Relative TP53 mRNA expression based on GEO database (GSE138198)

Note. GSE138198 data detected the expression of mRNAs in thyroid tissues of HT patients; Differentially expressed mRNAs in GSE138198 were identified using the limma package in R; n = 3 in normal samples*, n* = 13 in HT samples.
